# The mechanisms underlying the muscle metaboreflex modulation of sweating and cutaneous blood flow in passively heated humans

**DOI:** 10.14814/phy2.13123

**Published:** 2017-02-09

**Authors:** Baies Haqani, Naoto Fujii, Narihiko Kondo, Glen P. Kenny

**Affiliations:** ^1^ Human and Environmental Physiology Research UnitSchool of Human KineticsUniversity of OttawaOttawaCanada; ^2^ Laboratory for Applied Human PhysiologyGraduate School of Human Development and EnvironmentKobe UniversityKobeJapan

**Keywords:** Heat stress, metaboreceptors, skin blood flow, sudomotor activity, thermoregulation

## Abstract

Metaboreceptors can modulate cutaneous blood flow and sweating during heat stress but the mechanisms remain unknown. Fourteen participants (31 ± 13 years) performed 1‐min bout of isometric handgrip (IHG) exercise at 60% of their maximal voluntary contraction followed by a 3‐min occlusion (OCC), each separated by 10 min, initially under low (LHS, to activate sweating without changes in core temperature) and high (HHS, whole‐body heating to a core temperature increase of 1.0°C) heat stress conditions. Cutaneous vascular conductance (CVC) and sweat rate were measured continuously at four forearm skin sites perfused with 1) lactated Ringer's solution (Control), 2) 10 mmol L‐NAME [inhibits nitric oxide synthase (NOS)], 3) 10 mmol Ketorolac [inhibits cyclooxygenase (COX)], or 4) 4 mmol theophylline (THEO; inhibits adenosine receptors). Relative to pre‐IHG levels with Control, NOS inhibition attenuated the metaboreceptor‐mediated increase in sweating under LHS and HHS (*P* ≤ 0.05), albeit the attenuation was greater under LHS (*P* ≤ 0.05). In addition, a reduction from baseline was observed with THEO under LHS during OCC (*P *≤ 0.05), but not HHS (both *P* > 0.05). In contrast, CVC was lower than Control with L‐NAME during OCC in HHS (*P *≤ 0.05), but not LHS (*P* > 0.05). We show that metaboreceptor activation modulates CVC via the stimulation of NOS and adenosine receptors, whereas NOS, but not COX or adenosine receptors, contributes to sweating at all levels of heating.

## Introduction

In humans, the heat loss responses of cutaneous blood flow and sweating are important in the regulation of core temperature during heat stress. It is well documented that these responses can also be modulated by nonthermal muscle metaboreceptors (Crandall et al. 1998; Kondo et al. 1999; McGinn et al. 2014c; Amano et al. 2015, 2016; Paull et al. 2015). Specifically, metaboreflex activation, achieved by isometric handgrip (IHG) exercise followed by forearm ischemia to trap the produced metabolites, stimulates reductions in cutaneous vascular conductance (CVC) during hyperthermia, which is associated with a withdrawal of cholinergic vasodilator nerve activity (Crandall et al. 1998; Shibasaki et al. 2009). In addition, it has been well established that activation of metaboreceptors stimulates increases in sweating under heat stress (Crandall et al. 1998; Kondo et al. 1999, 2002; Shibasaki et al. 2001; Binder et al. 2012; McGinn et al. 2014c; Paull et al. 2015). While a proposed link between active vasodilation and sweating has been suggested (Kellogg et al. 1991, 1995; Vissing et al. 1991), some studies have suggested that cholinergic active vasodilation and sweating are independently controlled during IHG exercise and forearm ischemia (Crandall et al. 1995, 1998). However, the mechanisms underlying the heat loss responses during metaboreflex activation are still largely unknown.

Previous reports in humans in vivo have established that nitric oxide (NO) synthase (NOS), cyclooxygenase (COX), and/or adenosine (subtypes A_1_ and A_2_) receptors can mediate cutaneous vascular regulation during heat stress (Kellogg et al. 1998; Wilkins et al. 2003; Holowatz et al. 2005; McCord et al. 2006; Welch et al. 2009; Wong 2013; Fujii et al. 2014; McGinn et al. 2014a,b; McNamara et al. 2014; Swift et al. 2014). In addition to the role in cutaneous perfusion, NOS (Welch et al. 2009; Stapleton et al. 2014; Louie et al. 2016) and COX (Fujii et al. 2014, 2015) are integral components in the sweating response to heat stress, whereas studies have elucidated that adenosine receptors play a lesser role in the sweating response to heat stress (McGinn et al. 2014b; Swift et al. 2014). However, it is important to note that the level of influence of NOS, COX, and adenosine receptors to cutaneous blood flow and sweating can vary based on the type and level of heating (Kellogg et al. 1998; Shastry et al. 1998; McCord et al. 2006; Welch et al. 2009; Fieger and Wong 2012; Fujii et al. 2014; McGinn et al. 2014b; Swift et al. 2014; Louie et al. 2016). The relative contribution of these factors in the regulation of cutaneous perfusion and sweating during metaboreflex activation has yet to be elucidated.

Thus, in this study, we evaluated the involvement of NOS, COX, and adenosine receptors in the regulation of sweating and CVC during the activation of metaboreceptor (IHG followed by forearm ischemia) during whole‐body passive heating. We hypothesized that (1) NOS and COX, but not adenosine receptors, may play a role in the metaboreflex‐induced increases in sweating; and (2) NOS, COX, and adenosine receptors mediate the attenuation of CVC during the activation of metaboreceptors.

## Methods

### Ethical approval

This study was approved by the University of Ottawa Health Sciences and Science Research Ethics Board and conformed to the guidelines set forth by the Declaration of Helsinki. Written informed consent was obtained from all volunteers prior to their participation in the study.

### Participants

Fourteen healthy and physically active (exercise 2–3 times per week for 30 min in duration) males volunteered for the study. Participants had no history of smoking, cardiovascular disease, or autonomic disorders, and they were not taking prescription and/or over‐the‐counter medications at the time of the study. Mean (±SD) characteristics of the participants were: age, 31 ± 13 years; height, 1.77 ± 0.05 m; body mass, 79.5 ± 10.0 kg; and body surface area, 1.96 ± 0.13 m^2^.

## Experimental procedures

This study consisted of one experimental session. Participants were encouraged to abstain from taking vitamins and minerals at least 24 h prior to the session. They were asked to abstain from alcohol, caffeine, and strenuous physical activity 12 h before the session.

Participants reported to the laboratory on the day of the experimental session after eating a small meal no later than 2 h before their arrival. Shortly after their arrival, participants changed into shorts and provided a urine sample for the verification of their level of hydration. Urine specific gravity was measured using a hand‐held total solids refractometer (Model TS400, Reichter, Depew, NY). Afterward, body height and nude body mass were measured using a stadiometer and digital weight scale platform (model CBU150X, Mettler Toledo, Scherzenbach, Switzerland) with a weighing terminal (Model IND560, Mettler Toledo), respectively. Body surface area was subsequently determined using the measurements of body height and body mass (Dubois and DuBois 1916). Following instrumentation for the heart rate and body temperature measurements, participants donned a high‐density tube‐lined water perfusion garment (Allen‐Vanguard Corp., Ottawa, ON, Canada) that covered the entire body except the head, hands, feet, and forearms. Participants remained seated in a recumbent position on a bed for the remainder of the session in a thermoneutral environment (ambient temperature of 28°C).

After participants were seated, they performed two brief (<3 sec) maximal voluntary contractions (MVC) with their right hand using a handgrip dynamometer. The relative work load for the IHG exercise was determined using the highest of the two MVC's (60% of maximal voluntary contraction, average workload of 32 ± 4 kg).

After the determination of MVC, subjects remained resting during which time four microdialysis fibers (30 kDa cutoff, 10 mm membrane; MD 2000, Bioanalytical Systems, West Lafayette, IN) were inserted in the dermal layer of the skin on the dorsal side of the left forearm. To insert the fibers, a 25‐gauge needle was placed into the unanesthetized skin using aseptic technique and exited the skin 2.0–2.5 cm from the point of entry. The microdialysis fiber was then threaded through the lumen of the needle. The needle was withdrawn and the fibers were secured in place with surgical tape, each separated by at least ~4.0 cm. Following the fiber insertion (~15 min), each fiber was continuously perfused in a randomized fashion with: (1) lactated Ringer's solution (Control, Baxter, Deerfield, IL), (2) 10 mmol *N* ^G^‐nitro‐L‐arginine methylester (L‐NAME, Sigma‐Aldrich, St Louis, MO), a nonselective NOS inhibitor, (3) 10 mmol ketorolac (KETO, Sigma‐Aldrich), a nonselective COX inhibitor, or (4) 4 mmol theophylline (THEO, Sigma‐Aldrich), a nonselective competitive adenosine (A_1_/A_2_) receptor blocker. The concentrations for each of the agents were chosen based on previous research utilizing intradermal microdialysis in human skin (Holowatz et al. 2005; Kellogg et al. 2005; McCord et al. 2006; Fieger and Wong 2010, 2012; Fujii et al. 2014; McGinn et al. 2014a,b). Prior to the start of the metaboreceptor activation protocol (see below), each site was continuously perfused for a minimum of 75 min at a rate of 4 *μ*L∙min^−1^ using a micro‐infusion pump (Model 4004, CMA Microdialysis, Solna, Sweden) to ensure the establishment of each blockade. Additionally, the total amount of time (~90 min) following fiber insertion has been established to be sufficient for the resolution of any traumatic effect associated with the needle and/or fiber insertion (Hodges et al. 2009). Thereafter, the agents were perfused continuously throughout the protocol until the maximal cutaneous vasodilation protocol began (see below).

**Figure 1:**
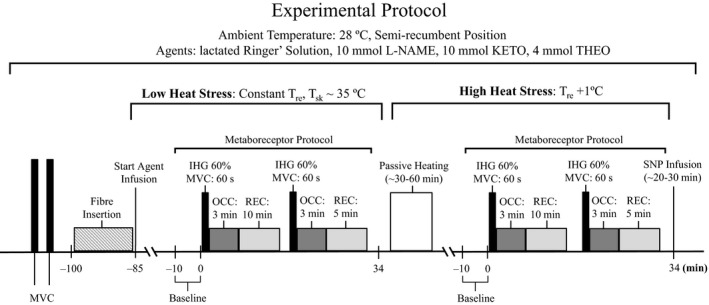
Schematic of the experimental protocol. Pharmacological agents employed are (1) lactated Ringer's solution; (2) 10 mmol *N*^G^‐nitro‐L‐arginine methylester (L‐NAME); (3) 10 mmol ketorolac (KETO); or (4) 4 mmol theophylline (THEO). MVC, maximal voluntary contraction; IHG, isometric handgrip exercise; OCC, forearm occlusion; REC, post‐ischemic recovery, Tre, rectal temperature; Tsk, mean skin temperature; SNP, sodium nitroprusside.

At the end of the 75‐min resolution period, the garment worn by the participants was perfused with water and maintained at 37.5°C (Low Heat Stress [LHS]) to stimulate eccrine sweating without inducing changes in core temperature prior to the start of the metaboreceptor activation protocol. This protocol was employed to ensure that a sweating response to metaboreceptor activation would be observed, given that previous reports showed no sweating response to the metaboreflex under normothermic conditions (i.e., when the sweat gland activation is insufficient) (Crandall et al. 1998; Kondo et al. 1999; Shibasaki et al. 2001; Binder et al. 2012). At the end of the 75‐min resolution period, baseline resting data were then collected for 10 min. This was followed by the completion of the metaboreceptor activation protocol which consisted of a 1‐min bout of IHG exercise at 60% of maximal voluntary contraction immediately followed by 3‐min of forearm blood flow occlusion (OCC), after which the occlusion was released and the participant recovered for 5 min (REC). The occlusion was achieved by inflating the pressure cuff to suprasystolic levels (>240 mmHg). This procedure was performed twice; each separated by a 10‐min interval. Thereafter, participants underwent a period of whole‐body heating to induce and maintain an increase in rectal temperature of 1.0°C (High Heat Stress [HHS]) above LHS levels. To achieve this, 49°C water was perfused through the garment and participants were covered with an insulated blanket to restrict heat loss to the environment. Upon completion of the heating period, the temperature of the water being perfused through the suit was reduced to maintain a steady‐state rectal temperature, which was followed by an additional ~20 min to ensure that the measurements had stabilized. Thereafter, a second 10‐min baseline was obtained, followed by the metaboreceptor activation protocol. See Figure 1 for a graphical representation of the experimental protocol.

At the end of the session, participants remained resting in the recumbent position while 50 mmol sodium nitroprusside (SNP; Sigma‐Aldrich) was perfused for ~20–30 min at a rate of 6 *μ*L∙min^−1^ at all sites to achieve maximal cutaneous vasodilation as defined by a plateau in cutaneous blood flow measurements for at least 2‐min. Blood pressure was measured at this time period to quantify cutaneous vascular conductance. A final nude body mass and urine sample were obtained at the end of the experimental protocol.

### Measurements

Ventilated plastic capsules (each covering an area of 1.1 cm^2^), designed for use with intradermal microdialysis (Meade et al. 2016), housed the laser‐Doppler flowmetry probes (see below). They were attached to the skin using adhesive rings, topical skin glue (Collodion HV, Mavidon Medical Products, Lake Worth, FL), and surgical tape directly over the microdialysis membranes to allow for the simultaneous measurement of local forearm sweat rates and skin blood flow at each skin site. Dry compressed air was supplied over the skin surface at a rate of 0.20 L∙min^−1^. High‐precision dew point mirrors (model 473, RH systems, Albuquerque, NM) were used to measure the water content from the effluent air. Local forearm sweat rate was calculated every 5 sec as the difference in water content between the effluent and influent air, multiplied by the flow rate, and normalized for the skin under the capsule, which was expressed in mg∙min^−1^∙cm^−2^mg∙min.

To obtain an index of skin blood blow, skin red blood cell flux (perfusion units) was measured locally at a sampling rate of 32 Hz using laser‐Doppler flowmetry (PeriFlux System 5000, Perimed AB, Stockholm, Sweden). Each flowmetry probe with a seven‐laser array (integrating probe 413, Perimed AB) was placed and secured directly over the microdialysis membranes throughout the duration of the experimental session. Cutaneous vascular conductance (CVC) was calculated as the perfusion units divided by mean arterial pressure (MAP; diastolic blood pressure plus one‐third of pulse pressure), and expressed as a percentage of maximum, as evaluated during the maximal cutaneous vasodilation procedure (%CVC_max_).

Rectal temperature was measured using a general purpose thermocouple probe, 2 mm in diameter (Mon‐a‐Therm; Mallinckrodt Medical, St. Louis, MO), inserted a minimum of 12 cm past the anal sphincter. Skin temperature measurements were obtained using thermocouples (Concept Engineering, Old Saybrook, CT) attached to four skin sites using surgical tape and a double‐sided adhesive ring. Mean skin temperature was calculated using the regional proportions determined by Ramanathan (1964): chest, 30%; biceps, 30%; quadriceps, 20%; and front calf, 20%. Temperature data were collected at a sampling rate of 15 s using an HP Agilent data acquisition module (model 3497A, Agilent Technologies Canada Inc., Mississauga, ON, Canada) and simultaneously displayed and recorded in spreadsheet format on a desktop computer with LabVIEW software (version 7.0; National Instruments, Austin, TX).

Mean arterial pressure and heart rate were measured continuously using a Finometer (Finapres Medical Systems, Amsterdam, The Netherlands). Measurements were obtained from the beat‐to‐beat recording of the left middle finger arterial pressure waveform via the volume‐clamp method (Penaz 1973). The Finometer was calibrated using physical criteria (Wesseling et al. 1995), and upper arm return‐to‐flow systolic pressure detection (Bos et al. 1996) following brachial artery pressure reconstruction (Gizdulich et al. 1996, 1997). Mean arterial pressure was verified periodically by manual auscultation using a validated mercury column sphygmomanometer (Baumanometer Standby model, WA Baum, Copiague, NY). Heart rate was verified using a Polar‐coded wearlink transmitter (Polar RS400 interface, and Polar Trainer 5 software, Polar Electro, Oy, Finland). A final blood pressure measurement was taken at the end of the maximal cutaneous vasodilation protocol.

### Data analysis

Prior to the metaboreceptor protocol, baseline resting values for the dependent variables of local sweat rate and CVC as well as rectal and mean skin temperatures were averaged over 1‐min prior to IHG exercise. To minimize the influence of an anticipatory response, a 1‐min average of both MAP and HR data were acquired 2‐min prior to the IHG exercise. All dependent variables were obtained by averaging the following time periods for both heat conditions (i.e., LHS and HHS): 15 sec of IHG exercise (local sweat rate for IHG exercise was the peak increase in sweat rate recorded during the metaboreceptor protocol), the final 15 sec of postexercise forearm occlusion, and the final 15 sec of recovery following the forearm occlusion. Additionally, rectal and mean skin temperatures were averaged during the metaboreceptor protocol for the final 30 sec of IHG exercise, the final 1‐min of postexercise forearm occlusion, and the final 1‐min of recovery from forearm occlusion. The change for all variables was calculated relative to pre‐IHG exercise baseline for the LHS and HSS conditions during the metaboreceptor protocols. The data for all variables during the two successive metaboreceptor activation protocols for each of the heating phases were combined as the responses did not differ.

### Statistical analysis

To compare responses between the four forearm skin treatment sites during the activation of metaboreceptors, a three‐way repeated‐measures ANOVA was performed for four skin sites (Control, L‐NAME, KETO, and THEO), under two different heat stress conditions (two levels: LHS and HHS) and as a function of the metaboreceptor protocol stage (IHG exercise, OCC, and REC) for sweating and CVC relative to pre‐IHG baseline levels. Thermal (i.e., rectal and mean skin temperatures) and cardiovascular variables (i.e., mean arterial pressure and heart rate) were analyzed using a two‐way repeated‐measures ANOVA with the factors of heating phase (two levels: LHS and HHS) and metaboreceptor protocol stage (four levels: baseline, IHG exercise and OCC, and REC). All values are presented as means ± SE unless otherwise noted. The level of significance for all analyses was set at an alpha level of *P* ≤ 0.05. All statistical analyses were completed using the software package SPSS 23 for Windows (IBM, Armonk, NY).

## Results

### Low heat stress

#### Hemodynamic responses

Cardiovascular responses for each heating phase are presented in Table 1. Relative to pre‐IHG baseline levels, MAP increased during IHG exercise and remained elevated during OCC (increase in MAP: IHG, 24 ± 2; OCC, 16 ± 2 mmHg; *P *≤ 0.05) and returned to baseline levels during REC (*P *> 0.05). Similarly, HR was elevated by 28 ± 3 beats∙min^−1^ from pre‐IHG baseline levels during IHG (*P *≤ 0.05) and returned to pre‐IHG baseline levels during OCC and REC (both *P *> 0.05).

**Table 1:** Cardiovascular and thermal responses at baseline, end isometric handgrip exercise, forearm occlusion, and recovery under the low and high heat stress conditions

	BL	IHG	OCC	REC
	Low heat stress			
MAP, mmHg	89 ± 2	113 ± 3^a^	105 ± 2^a^	90 ± 2
HR, beats∙min^−1^	67 ± 3	95 ± 5^a^	67 ± 3	66 ± 3
T_re_,˚C	37.14 ± 0.06	37.14 ± 0.06	37.13 ± 0.06	37.13 ± 0.06
T_sk_, ˚C	35.17 ± 0.12	35.17 ± 0.12	35.22 ± 0.12^a^	35.18 ± 0.11
	High heat stress			
MAP, mmHg	92 ± 2	114 ± 2^a^	106 ± 2^a^	94 ± 2^a^
HR, beats∙min^−1^	99 ± 4	120 ± 6^a^	99 ± 4	97 ± 4
T_re_, °C	38.17 ± 0.11	38.17 ± 0.11	38.14 ± 0.11^a^	38.12 ± 0.11^a^
T_sk_,°C	36.36 ± 0.09	36.36 ± 0.09	36.38 ± 0.10	36.36 ± 0.10

Values are presented as means ± SE. MAP, mean arterial pressure; HR, heart rate; Tre, rectal temperature; Tsk, mean skin temperature; BL, baseline; IHG, end isometric handgrip exercise; OCC, forearm occlusion; REC, recovery.

a Significantly different from BL (*P *≤ 0.05).

#### Thermal responses

Thermal responses for each heating phase are presented in Table 1. T_sk_ was elevated by 0.05 ± 0.01°C during OCC from pre‐IHG baseline (*P* ≤ 0.05). T_re_ did not change from pre‐IHG baseline levels during the metaboreceptor protocol (*P* > 0.05).

#### Cutaneous vascular response

**Figure 2:**
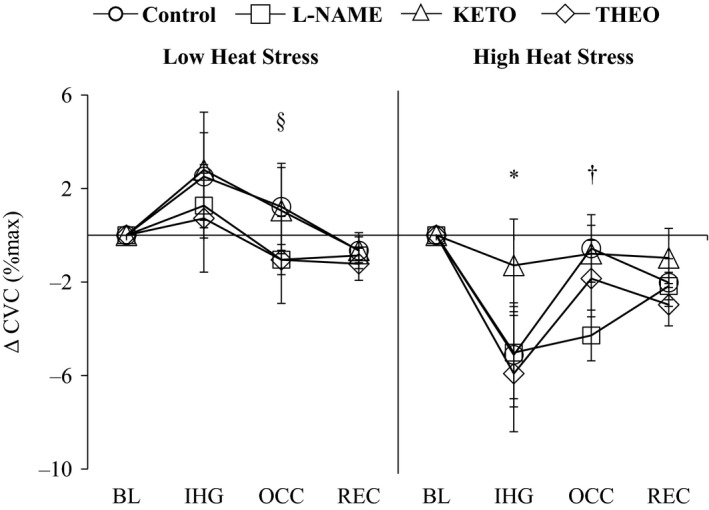
The change (∆) in CVC relative to BL at the end IHG exercise, OCC, and REC under low heat stress and high heat stress conditions at each treatment site. Pharmacological agents employed are (1) lactated Ringer's solution (Control, circles); (2) 10 mmol *N*^G^‐nitro‐L‐arginine methylester (L‐NAME, squares), a nonselective NOS inhibitor; (3) 10 mmol ketorolac (KETO, triangles), a nonselective COX inhibitor; or (4) 4 mmol theophylline (THEO, diamonds), a nonselective competitive adenosine (A_1_/A_2_) receptor inhibitor. Values are means ± SE. *significantly different from BL for Control, L‐NAME, and THEO (*P* ≤ 0.05); ^†^Control different from L‐NAME (*P* ≤ 0.05); ^§^Control different from THEO (*P* ≤ 0.05). NOS, nitric oxide synthase; COX, cyclooxygenase; CVC, cutaneous vascular conductance; BL, Baseline; IHG, isometric handgrip exercise; OCC, occlusion; REC, post‐ischemic recovery.

During pre‐IHG baseline, CVC did not change with KETO relative to Control (Table 2; *P *> 0.05); however, CVC was lower and higher than Control for L‐NAME and THEO, respectively (*P* ≤ 0.05) (Pre‐IHG baseline levels: Control, 24.1 ± 3.0; L‐NAME, 13.7 ± 1.6; KETO, 28.5 ± 3.4; THEO, 35.1 ± 4.3% CVC maximum). IHG exercise and OCC did not alter CVC relative to pre‐IHG levels (Fig. 2; *P *> 0.05), but the relative change in CVC was reduced during OCC from Control for THEO only (Fig. 2; *P* ≤ 0.05). All sites remained similar to baseline during REC (*P *> 0.05).

**Table 2:** Local forearm sweat rate and cutaneous vascular conductance at baseline, end isometric handgrip exercise, forearm occlusion, and recovery under the low and high heat stress conditions

	BL	IHG	OCC	REC
	Low heat stress			
SR, mg∙min^−1^∙cm^−2^				
Control	0.30 ± 0.03	0.58 ± 0.06^a^	0.55 ± 0.06^a^	0.32 ± 0.04
L‐NAME	0.31 ± 0.02	0.52 ± 0.05^a^	0.50 ± 0.06^a^	0.33 ± 0.03
KETO	0.29 ± 0.02	0.59 ± 0.05^a^	0.53 ± 0.05^a^	0.30 ± 0.02
THEO	0.32 ± 0.06	0.60 ± 0.05^a^	0.57 ± 0.06^a^	0.35 ± 0.04
CVC, %maximum				
Control	24.1 ± 3.0	26.6 ± 3.2	25.3 ± 3.1	23.4 ± 3.2
L‐NAME	13.7 ± 1.6^b^	14.9 ± 2.1^b^	12.6 ± 1.6^b^	12.8 ± 1.4^a^^,^ b
KETO	28.5 ± 3.4	31.3 ± 4.3	29.5 ± 3.8	27.8 ± 3.6
THEO	35.1 ± 4.3^b^	35.8 ± 3.6^b^	34.1 ± 4.0^b^	33.9 ± 4.5^b^
	High heat stress			
SR, mg∙min^−^ b∙cm^−2^				
Control	1.66 ± 0.16	1.70 ± 0.16^a^	1.70 ± 0.16^a^	1.64 ± 0.18
L‐NAME	1.54 ± 0.12	1.57 ± 0.12^a^	1.57 ± 0.12^a^	1.53 ± 0.13
KETO	1.75 ± 0.14	1.80 ± 0.14^a^	1.81 ± 0.14^a^	1.74 ± 0.15
THEO	1.63 ± 0.15	1.68 ± 0.15^a^	1.68 ± 0.15^a^	1.62 ± 0.17
CVC, %maximum				
Control	66.0 ± 4.0	60.9 ± 4.5^a^	65.5 ± 4.8	64.0 ± 4.0
L‐NAME	39.2 ± 3.6^b^	34.2 ± 3.7^a^, ^b^	34.9 ± 3.4^a^, ^b^	37.1 ± 3.7^a^^,^ b
KETO	61.1 ± 4.4	59.8 ± 4.7	60.3 ± 4.2	60.1 ± 4.3
THEO	69.9 ± 3.7	64.0 ± 4.3^a^	68.1 ± 3.8	67.0 ± 3.8^a^

SR, sweat rate; CVC, cutaneous vascular conductance; BL, baseline; IHG, end isometric handgrip exercise; OCC, forearm occlusion; REC, recovery.

Pharmacological agents employed are (1) lactated Ringer's solution (Control); (2) 10 mmol *N* ^G^‐nitro‐L‐arginine methylester (L‐NAME); (3) 10 mmol ketorolac (KETO); or (4) 4 mmol theophylline (THEO).

Values are presented as means ± SE.

a Significantly different from BL (*P *≤ 0.05).

b Significantly different from Control (*P *≤ 0.05).

#### Sweating

**Figure 3:**
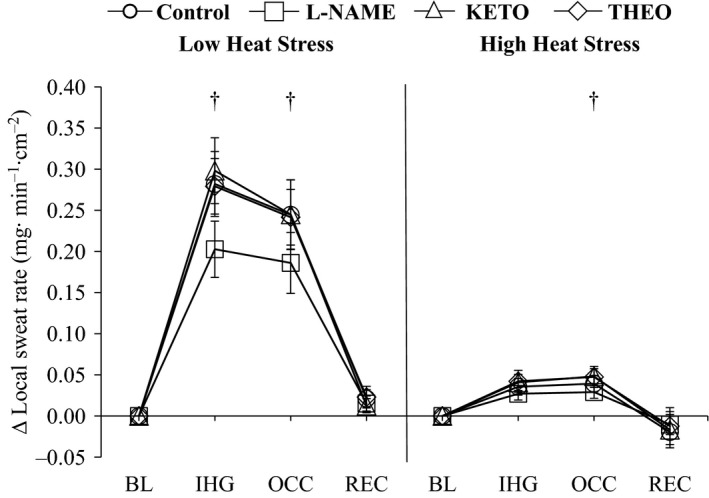
The change (∆) in local sweat rate relative to BL at the end IHG exercise, OCC, and REC under low heat stress and high heat stress at each treatment site. Pharmacological agents employed are (1) lactated Ringer's solution (Control, circles); (2) 10 mmol *N*^G^‐nitro‐L‐arginine methylester (L‐NAME, squares), a nonselective NOS inhibitor; (3) 10 mmol ketorolac (KETO, triangles), a nonselective COX inhibitor; or (4) 4 mmol theophylline (THEO, diamonds), a nonselective competitive adenosine (A_1_/A_2_) receptor inhibitor. Values are means ± SE. *significantly different from BL for all sites (*P* <* *0.05); ^†^Control different from L‐NAME (*P* < 0.05). NOS, Nitric Oxide Synthase; COX, cyclooxygenase; BL, baseline; IHG, isometric handgrip exercise; OCC, occlusion; REC, post‐ischemic recovery.

Local sweat rates were comparable between sites during pre‐IHG baseline (Table 2; *P* > 0.05). Sweat rate for all sites was elevated during both the IHG exercise and OCC periods (Fig. 3; *P* ≤ 0.05). The magnitude of increase in sweating differed between sites such that a ~20 ± 12% reduction in sweating during IHG exercise with L‐NAME was observed relative to Control (*P* ≤ 0.05). The reduction was maintained by ~25 ± 9% during OCC (*P* ≤ 0.05).

### High heat stress

#### Hemodynamic responses

IHG exercise resulted in an increase in MAP by 22 ± 1 mmHg, relative to pre‐IHG baseline. During OCC, MAP remained elevated above baseline (*P* ≥ 0.05). Further, IHG exercise increased HR significantly above pre‐IHG baseline by 21 ± 3 beats∙min^−1^ (*P *≤ 0.05). During OCC, HR returned to baseline levels and was maintained at pre‐IHG baseline levels during REC (*P *≥ 0.05).

#### Thermal responses

T_sk_ was maintained at stable levels at ~36.3°C during the metaboreceptor protocol (*P* > 0.05). T_re_ was increased to 1°C above LHS at pre‐IHG levels. However, T_re_ was reduced from pre‐IHG baseline by 0.05 ± 0.01°C when compared with REC (*P* ≤ 0.05).

#### Cutaneous vascular response

During pre‐IHG baseline, L‐NAME attenuated CVC from Control (Table 2; *P *≤ 0.05). Conversely, KETO and THEO were similar to Control (*P* > 0.05). All sites exhibited a ~5–6% CVC decrease during IHG exercise (Fig. 2; *P *≤ 0.05), whereas KETO remained unchanged from baseline levels (*P* > 0.05) and tended to be elevated from Control (*P *= 0.06). However, during OCC, both Control and THEO returned back to pre‐IHG baseline levels (both *P* > 0.05), while L‐NAME remained attenuated to levels lower than Control (*P* ≤ 0.05).

#### Sweating

During pre‐IHG baseline, local sweat rates were similar at all sites (Table 2; Pre‐IHG baseline levels: Control, 1.66 ± 0.16; L‐NAME, 1.54 ± 0.12; KETO, 1.75 ± 0.14; THEO, 1.63 ± 0.15 mg∙min^−1^∙cm^−2^; *P* > 0.05). All sites exhibited an increase in sweating above pre‐IHG baseline levels during both the IHG exercise and OCC periods (Fig. 3; *P* ≤ 0.05). The magnitude of increase was similar during IHG exercise between all sites (*P* > 0.05), whereas during OCC, sweating was reduced with L‐NAME only (*P* ≤ 0.05). Similar levels of sweating were observed thereafter during REC (all *P* > 0.05).

### LHS versus HHS

#### Hemodynamic responses

Pre‐IHG baseline MAP was similar under both heating phases (*P* ≥ 0.05), whereas, HR was significantly elevated during HHS from LHS (*P* ≤ 0.05). However, a greater elevation of MAP was observed with IHG than OCC under LHS than HHS (*P *≤ 0.05).

#### Sweating

Under HHS, sweating was elevated from LHS at all sites during pre‐IHG baseline (*P* ≤ 0.05). Although the pattern of response between heating phases were similar, the magnitude of increase was reduced under HSS relative to LHS during IHG exercise and OCC (*P* ≤ 0.05).

## Discussion

To the best of our knowledge, this is the first study to examine the mechanisms mediating the metaboreflex‐induced changes in cutaneous vascular and sweating responses during heat stress. We demonstrate that adenosine receptors play a role in modulating CVC during metaboreceptor activation under low, but not high heat stress conditions. While no involvement of COX was observed, we exhibit that NOS plays an important role in the metaboreceptor‐mediated modulation of cutaneous perfusion under high heat stress conditions only. We also show that NOS contributes to the metaboreflex‐mediated increase in sweating during low heat stress; albeit its contribution is diminished under high heat stress.

### Cutaneous vascular response

We demonstrate that NOS inhibition reduced CVC from Control during baseline resting under low and high heat stress (Table 1) which is a finding consistent with previous literature (Kellogg et al. 1998; Wilkins et al. 2003; Stanhewicz et al. 2013; Wong 2013; Swift et al. 2014). Moreover, we detected a vasoconstrictor effect of adenosine receptors on baseline CVC under low heat stress (Fieger and Wong 2012; McGinn et al. 2014a,b; Swift et al. 2014), whereas we illustrate no adenosine receptor‐mediated component to CVC during high levels of heating at baseline. However, in contrast to the findings of McCord et al. (2006), we did not observe a COX‐dependent vasodilation as defined by an attenuation of CVC during heat stress with COX inhibition. The underlying reason for this disparity is unclear and requires further examination. Noteworthy, the pattern of response measured at baseline remained intact for the duration of the metaboreceptor protocol during both low and high heat stress condition. Future studies are warranted to decipher this observation.

Consistent with previous reports, we confirm that IHG exercise and post‐IHG ischemia does not modulate CVC under low levels of heating at the Control site (Crandall et al. 1998; Kondo et al. 2003; McCord and Minson 2005; Binder et al. 2012). We measured a similar pattern of response in CVC at the NOS‐inhibited site during the postischemic period. This observation indicates that under low levels of heating NOS does not contribute to the regulation of cutaneous blood flow during the activation of metaboreceptors. In contrast to what we observed under low heat stress, a decrease in CVC at the Control site occurred relative to baseline during IHG exercise, albeit CVC returned to near baseline levels during the subsequent period of ischemia under the high heat stress condition (i.e., >0.8°C) (Binder et al. 2012) (Fig. 2). Accordingly, several reports (Crandall et al. 1998; Kondo et al. 2003; Binder et al. 2012) demonstrate that the metaboreceptor modulation of cutaneous blood flow differs with increases in the level of heat stress; a response we observed in this study. However, a finding unique to this study is that during high heat stress conditions, CVC was reduced during post‐IHG ischemia with the administration of a NOS antagonist relative to the Control site; a response that demonstrates an important role of NOS in the regulation of cutaneous blood flow during metaboreflex activation. The reduction in CVC during the metaboreflex observed at Control under heat stress has been attributed to both neural (i.e., withdrawal of cholinergic vasodilator nerve activity) (Crandall et al. 1998; Shibasaki et al. 2009) and non‐neural (i.e., through input of local mediators) (McCord and Minson 2005; Shibasaki et al. 2009) mechanisms. Further, it has been established that changes in shear stress (as indicated by changes in perfusion and/or pressure) can modulate NO production (Kuchan and Frangos 1994; Vequaud and Freslon 1996; Paniagua et al. 2001). Given our observation, a NOS‐dependent attenuation in CVC and a rise in mean arterial pressure during the metaboreflex, changes in CVC with the NOS blockade may be manifested via changes in vascular shear stress. Taken together, our findings may indicate that changes in NO availability induced by changes in vascular perfusion/pressure associated with the metaboreflex may be a key factor in the regulation of CVC during high heat stress. While this suggests that the NOS‐mediated contribution occurs through input of local mediators, a centrally mediated effect cannot be discounted.

While no COX‐dependent contribution was measured under low heat stress during IHG exercise and post‐IHG ischemia, COX inhibition tended to blunt the reduction in CVC during IHG exercise under high heat stress. However, the precise mechanism (neural vs. non‐neural) mediating the involvement of COX‐derived prostanoid(s) during IHG exercise cannot be elucidated and requires further scrutiny. Further, in contrast to NOS, no COX‐dependent contribution on the regulation of CVC was measured during the postexercise ischemic period under high heat stress (Fig. 2). Consequently, given that a role of COX was not confirmed during the period of ischemia, factors unrelated to metaboreceptors may be implicated in modulating COX activity during high heat stress.

We detected a lower CVC with adenosine receptor blockade as compared with Control during the metaboreflex under lower levels of heating only (Fig. 2). This indicates that adenosine receptors play a role in dilating cutaneous vessels in this situation. Regarding possible mechanisms, McCord and Minson (2005) indicated that greater level of dilation of the blood vessel is required to observe the cutaneous vascular response to IHG exercise. This response may therefore explain our observation of an adenosine receptor‐dependent reduction in CVC. Also, this is supported by our observation that adenosine receptor blockade with THEO resulted in higher baseline CVC during the occlusion period.

### Sweating response

An early report by Kellogg et al. (1998) showed no role for NOS in the regulation of sweating under passive heating conditions eliciting low‐to‐moderate increases in sweat rate (i.e., ~0.4 mg∙min^−1^∙cm^−2^). We extend upon this work by demonstrating that this response remains unchanged at baseline under high heat stress conditions that are associated with very high sweat rates (~1.6 mg∙min^−1^∙cm^−2^). Similarly, we show that COX or adenosine receptors are not involved in the regulation of sweating under low heat stress conditions. Further, this pattern of response remains intact under high heat stress conditions via whole‐body passive heating which are findings unique to this study.

Muscle metaboreceptor activation has been shown to augment sweating from baseline levels (as evaluated during the post‐IHG ischemia) under conditions where sweat glands are activated (Crandall et al. 1998; Kondo et al. 1999). As we show for the first time, this response is NOS‐dependent and is manifested under both low and high levels of hyperthermia. The latter observation is particularly noteworthy given that the metaboreceptor‐induced increases in sweating are attenuated under conditions associated with elevated sweat production (i.e., high heat stress) (Binder et al. 2012). Our results indicate that NOS remains an important mediator in the metaboreflex modulation of sweating under high heat stress. It is important to note that while we observed differences in sweat rate between with NOS inhibition from Control under High heat stress the difference may not be physiologically relevant and would likely result in a negligible influence on whole‐body heat dissipation and therefore core temperature regulation. Lastly, in contrast to CVC, it appears that the mechanisms mediating the metaboreceptor‐induced elevation in the sweating response are similar under both low and high heating conditions, albeit the magnitude of contribution differs.

In contrast to our observation with NOS, we showed no involvement of COX or adenosine under low or high heat stress. Noteworthy, an increasing number of studies demonstrate that NOS and COX, but not adenosine, are key in modulating sweating during dynamic exercise (Welch et al. 2009; Fujii et al. 2014, 2015, 2016; McGinn et al. 2014b; Stapleton et al. 2014; Louie et al. 2016). In the context of our current findings, it is plausible therefore that the NOS‐mediated regulation of sweating during dynamic exercise may be subject to the influences of nonthermal metaboreflexes. Given that we did not observe a metaboreceptor‐induced effect on the relative contribution of COX on the sweating response, it can be surmised that COX is likely not influenced by this same nonthermal stimuli. Similarly, given that no effect of adenosine receptors on sweating was observed during and following exercise (McGinn et al. 2014b), along with our findings, it appears that adenosine receptors do not play a role in sweat production.

### Perspectives and significance

While it is well documented that nonthermal factors such as those associated with the activation of muscle metaboreceptors can influence the regulation of heat loss responses, we show that this involves the contribution of specific mechanisms. Importantly, a recent report by Amano et al. (2016) studied the contribution of the metaboreflex on the heat loss responses during dynamic exercise and demonstrated that the activation of metaboreceptors augments sweating but does not affect cutaneous vascular responses. However, this response differed from their previous observations during passive heating (Amano et al. 2015). In the context of our findings, our study informs the possible mechanisms mediating this response during exercise. In particular, we show that this response involves NOS. However, it is possible that other mechanisms may be at play. As such, future studies are necessary to extend upon this work. Additionally, further studies are required to assess these mechanism(s) for a longer duration of metaboreceptors activity (Amano et al. 2015, 2016) and or intensity/duration of IHG exercise paradigms (Crandall et al. 1995, 1998; Kondo et al. 2000, 2003; Shibasaki et al. 2001). All of the aforementioned factors may influence the level of metaboreflex activation and therefore the relative contribution of these different mediators.

In conclusion, we show that inhibition of adenosine receptors can modulate the CVC response to the metaboreflex under low levels of hyperthermia, whereas its influence is less evident under elevated heat stress conditions. In addition, we show that a COX‐dependent modulation of CVC is not evident during the activation of metaboreceptors, whereas NOS is involved in mediating cutaneous vasculature during the activation of muscle metaboreceptors under high levels of hyperthermia. Moreover, we demonstrate that NOS, but not COX or adenosine receptors, contributes to the sweating response during metaboreceptor activation under all levels of heating, albeit its contribution is diminished under high heat stress.

## Conflict of Interest

No conflicts of interest to declare.
